# Endothelial Cell Focal Adhesion Regulates Transendothelial Migration and Subendothelial Crawling of T Cells

**DOI:** 10.3389/fimmu.2018.00048

**Published:** 2018-01-24

**Authors:** Jaehyun Lee, Kwang Hoon Song, Taeyeob Kim, Junsang Doh

**Affiliations:** ^1^School of Interdisciplinary Bioscience and Bioengineering (I-Bio), Pohang University of Science and Technology (POSTECH), Pohang, South Korea; ^2^Department of Mechanical Engineering, Pohang University of Science and Technology (POSTECH), Pohang, South Korea

**Keywords:** leukocyte adhesion cascade, T cell, transendothelial migration, subendothelial crawling, endothelial cell, focal adhesion

## Abstract

Leukocytes circulating in the blood stream leave out of blood vessels and infiltrate into inflamed tissues to perform immune responses. Endothelial cells (ECs) lining interior of the post-capillary venules regulate various steps of leukocyte extravasation. In response to inflammatory signals, ECs upregulate adhesion molecules and produce/present chemokines to support firm adhesion and intraluminal crawling of leukocytes. They also remodel junctions to facilitate leukocyte transendothelial migration (TEM). While roles of apical/lateral components of EC layers in regulating leukocyte extravasation have been extensively investigated, relatively little attention has been paid to the basal part of EC layers comprising subendothelial spaces. In this study, we employed interference reflection microscopy (IRM), a microscopy technique specialized for label-free visualization of cell–substrate contact, to study detailed dynamic interactions between basal part of ECs and T cells underneath EC monolayer. For TEM, T cells on EC monolayer extended protrusions through junctions to explore subendothelial spaces, and EC focal adhesions (EC-FAs) acted as physical barrier for the protrusion. Therefore, preferential TEM occurred through junctions where near-junction focal adhesion (NJ-FA) density of ECs was low. After TEM, T cells performed subendothelial crawling (SEC) with flattened morphology and reduced migration velocity due to tight confinement. T cell SEC mostly occurred through gaps formed in between EC-FAs with minimally breaking EC-FAs. Tumor necrosis factor-α (TNF-α) treatment significantly loosened confinement in subendothelial spaces and reduced NJ-FA density of ECs, thus remodeled basal part of EC layer to facilitate leukocyte extravasation.

## Introduction

Circulating blood leukocytes infiltrate into inflamed tissues to eliminate the inflammatory triggers and mediate tissue repair (1, 2). For extravasation and tissue infiltration, leukocytes undergo sequential steps of dynamic interactions with endothelial cells (ECs) and other components in blood vessels, known as leukocyte adhesion cascade (3–6). Leukocyte adhesion cascade is initiated by EC activation by pro-inflammatory cytokines [e.g., tumor necrosis factor-α (TNF-α) and interleukin-1β (IL-1β)], which results in adhesion molecule upregulation and chemokine production. In response to EC activation, leukocytes in blood stream roll for a while (rolling), make firm adhesion on ECs (arrest), crawl on luminal surfaces of EC layers with polarized shape [intraluminal crawling (ILC)], and trans-migrate through the EC layers [transendothelial migration (TEM)]. Leukocytes successfully performed TEM crawl substantial distances in subendothelial spaces formed between an EC layer and pericytes/basement membrane [subendothelial crawling (SEC)] to breach basement membrane and eventually leave out of blood vessels to infiltrate into inflamed tissues.

Each step of the leukocyte adhesion cascade is regulated by various biochemical/biophysical cues in inflamed blood vessels. For example, E- and P-selectins are critical for rolling whereas intercellular cell adhesion molecules (ICAMs) and vascular cell adhesion molecules (VCAMs), ligands for leukocyte integrins, are essential for arrest, ILC, and TEM. Shear flow facilitates stable ILC and TEM in concert with chemokines (7–10). Spatial heterogeneity of biochemical/biophysical cues in inflamed blood vessels induces polarized migration of leukocytes. For example, leukocytes sense and response to physical properties of ECs, such as topography (11) and stiffness (12, 13) as well as chemokine gradient (14, 15), to steer directions for ILC, which is a critical step searching for preferential sites in luminal surfaces for TEM. After completing TEM, leukocytes explore subendothelial spaces by performing SEC, and preferentially breach low-expression region (LER) of the basement membrane where expression levels of matrix proteins are low (16–19). Optimal pathway finding in luminal and subendothelial spaces is critical for the successful extravasation; prolonged ILC may lead to detachment by shear flow (11), whereas prolonged SEC may increase probability of reverse TEM, or trans-migration of leukocyte in subendothelial spaces back to luminal spaces (20). While various factors regulating ILC, including adhesion molecules, chemokines, shear flow, mechanical properties of ECs, has been identified (3, 5, 21), how microenvironments in subendothelial spaces direct SEC has not been completely understood.

Subendothelial spaces in post-capillary venules are formed in between an EC layer and an incomplete layer of pericytes embedded in basement membrane (4). ECs are attached on pericytes/basement membrane layer by forming integrin-mediated focal adhesions (FAs) on basement membrane and N-cadherin-mediated adherens junctions with pericytes (22). EC adhesion may play an important role in later steps of the adhesion cascade, such as TEM and SEC, but such possibility has not been addressed. In this study, we exploited interference reflection microscopy (IRM), a label-free imaging technique specialized for the visualization of cell–substrate adhesion (23–27), to visualize leukocyte–EC adhesion interaction dynamics during extravasation. We demonstrated that EC focal adhesions (EC-FAs) acted as physical barriers for T cells so that TEM preferentially occurred through the junctions where EC-FA density was low, and SEC mostly occurred through the gaps formed between EC-FAs with minimally breaking EC-FAs.

## Results

### Label-Free Identification of FAs in EC Monolayers by IRM

**Figure 1:**
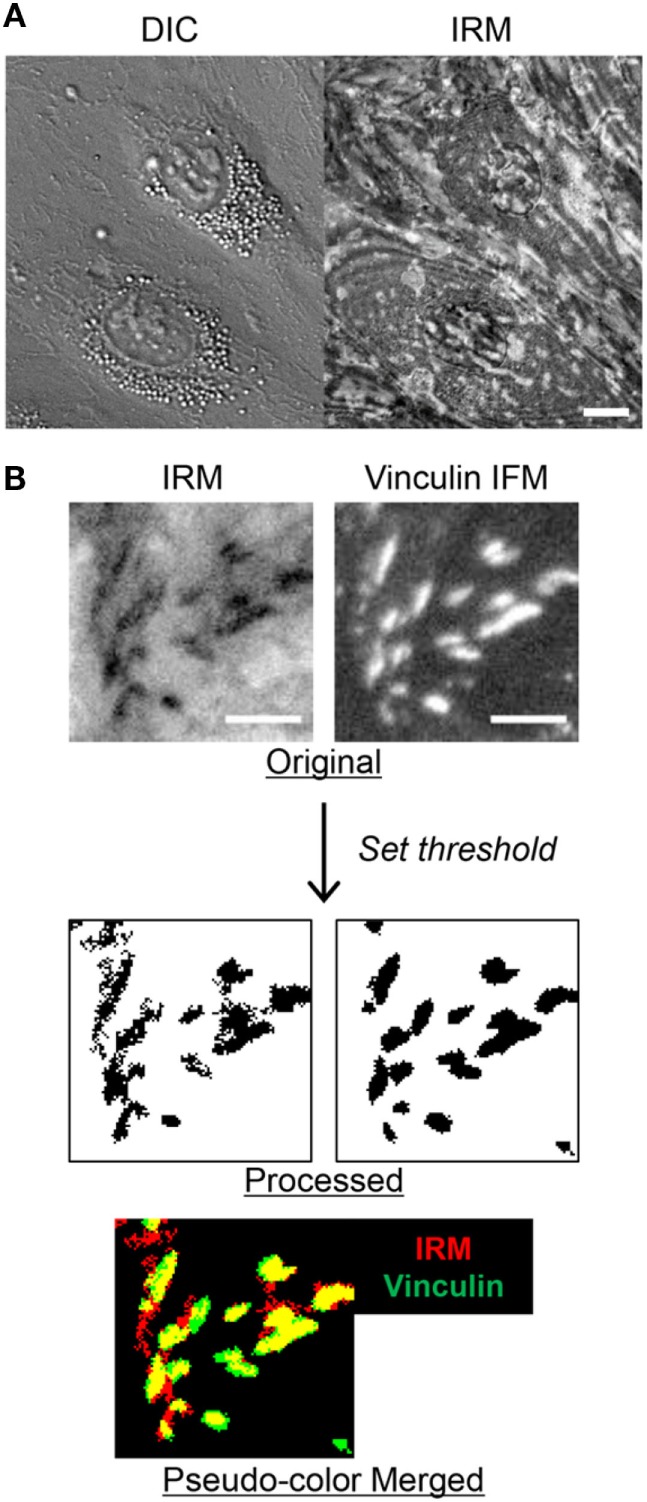
Identification of focal adhesions (FAs) in interference reflection microscopy (IRM) images of endothelial cell (EC) monolayer. **(A)** Differential interference contrast (DIC) and IRM images of EC monolayer. Scale bar: 10 µm. **(B)** Comparison of IRM and vinculin immunofluorescence microscopy (IFM) images of EC monolayer. In pseudo-color merged image, yellow regions represent overlapped regions of dark spots in IRM and bright spots in vinculin IFM images. Scale bar: 5 µm.

To visualize EC adhesion, we employed IRM, which generates gray-scale patterns depending on substrate–cell membrane proximity. In IRM images, lights reflect from basal cell membrane and the surface of the substrate generate interference patterns: typically, black spots in IRM images mean that basal cell membrane is located within 15 nm of distance from the substrate, whereas gray spots appear when the distance between basal cell membrane and substrate is between 15 and 100 nm (23–26). IRM images of EC monolayers revealed spatially heterogeneous adhesion pattern formation underneath EC monolayer (Figure 1A). We hypothesized that the dark spots in IRM images were FAs of ECs. Indeed, previous studies demonstrated that dark spots in IRM images coincided with FAs in many different cell types, including fibroblasts (25), embryo cells (24), and osteosarcoma cells (28). To test this hypothesis, we visualized vinculins, one of the main components of mature FAs, in fixed EC monolayer by immunofluorescence microscopy (IFM) in conjunction with IRM. Overall, dark spots in IRM images well matched with bright spots in vinculin IFM images (Figure 1B). To quantitatively test co-localization of dark spots in IRM images and vinculin IFM images, dark spots in IRM images and bright spots in vinculin IFM images were extracted by setting thresholds (“Processed” in Figure 1B) and compared. Threshold value for IRM images was manually determined to include the majority of apparent dark spots. When overlaid with pseudo-colors (“Pseudo-color Merged” in Figure 1B), 90.06 ± 0.99% of dark spots in IRM images overlapped with bright spots in vinculin IFM images. In addition, Pearson’s correlation coefficient, widely used for co-localization analysis (29), was 0.89 ± 0.02, meaning that dark spots in IRM images highly co-localized with bright spots in vinculin IFM images. Therefore, dark spots in IRM images of EC monolayer could be considered EC-FAs.

### TNF-α Treatment Altered EC Adhesion Patterns

**Figure 2:**
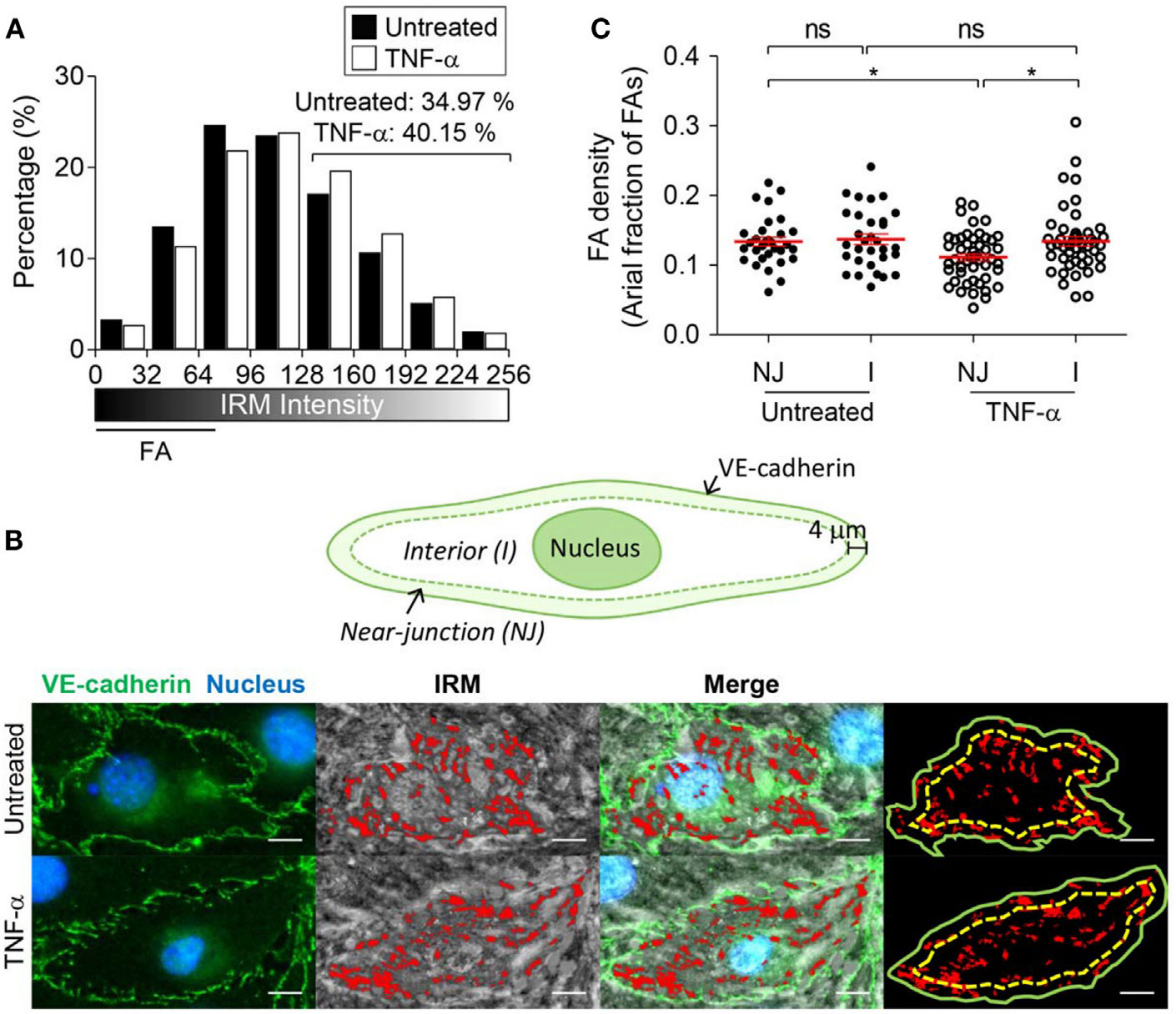
The effect of tumor necrosis factor-α (TNF-α) treatment on EC basal membrane adhesion and FA distribution. **(A)** IRM image intensity distributions of TNF-α untreated and treated EC monolayer (two-sample Kolmogorov–Smirnov test, *p* < 0.0001). **(B)** Definition of near-junction (NJ) and interior (I) regions (top panel), and identification of NJ-FA and I-FA using merged images of VE-cadherin IFM and IRM images (bottom panel). **(C)** EC-FA density in NJ or interior (I) regions in the presence or absence of TNF-α treatment (Mann–Whitney *U*-test, *p < 0.05, ns, not significant).

Next, we examined whether EC adhesion patterns were influenced by TNF-α, one of the major cytokines produced during inflammation. While the effects of TNF-α on EC activation, including adhesion molecule upregulation (30–32), junction remodeling (33, 34), and cytokine/chemokine production (35, 36), have been extensively studied, how TNF-α alters EC adhesions has not been reported. We first treated ECs with 10 ng/ml of TNF-α for 4 h, which is sufficient to induce substantial upregulation of adhesion molecules such ICAM-1, VCAM-1, P-selectin, and E-selectin (37). Then, IRM images of TNF-α-untreated and treated EC monolayer were acquired, and IRM intensities of each case, which corresponds to distance between EC membrane and substrate, were compared by histogram analysis (Figure 2A). TNF-α treatment significantly increased portions of high IRM intensities, meaning that overall cell–substrate contact was reduced by TNF-α.

TNF-α treatment can also alter FA distribution within EC monolayer. In particular, we focused on FA distribution near adherens junction regions, through which leukocyte paracellular TEM occurs. Adherens junctions of EC monolayer were visualized by fluorophore-conjugated antibody for VE-cadherin, and FA distribution with respect to the adherens junctions was analyzed by overlaying IRM and VE-cadherin IFM images (Figure 2B). Regions within 4 µm distance from VE-cadherin were defined as “near-junction (NJ)” regions, and the rest of regions were defined as “interior (I)” regions. Areal fraction of FAs in each region for individual ECs was measured and plotted (Figure 2C). While untreated ECs exhibited similar levels of FAs in NJ and I regions, TNF-α treatment significantly reduced areal fraction of FAs in NJ regions. Taken together, EC monolayer activated by TNF-α increased distance between EC basal membranes and substrate, and reduced NJ-FA density.

### Label-Free Imaging of T Cell Adhesion Cascades

**Figure 3:**
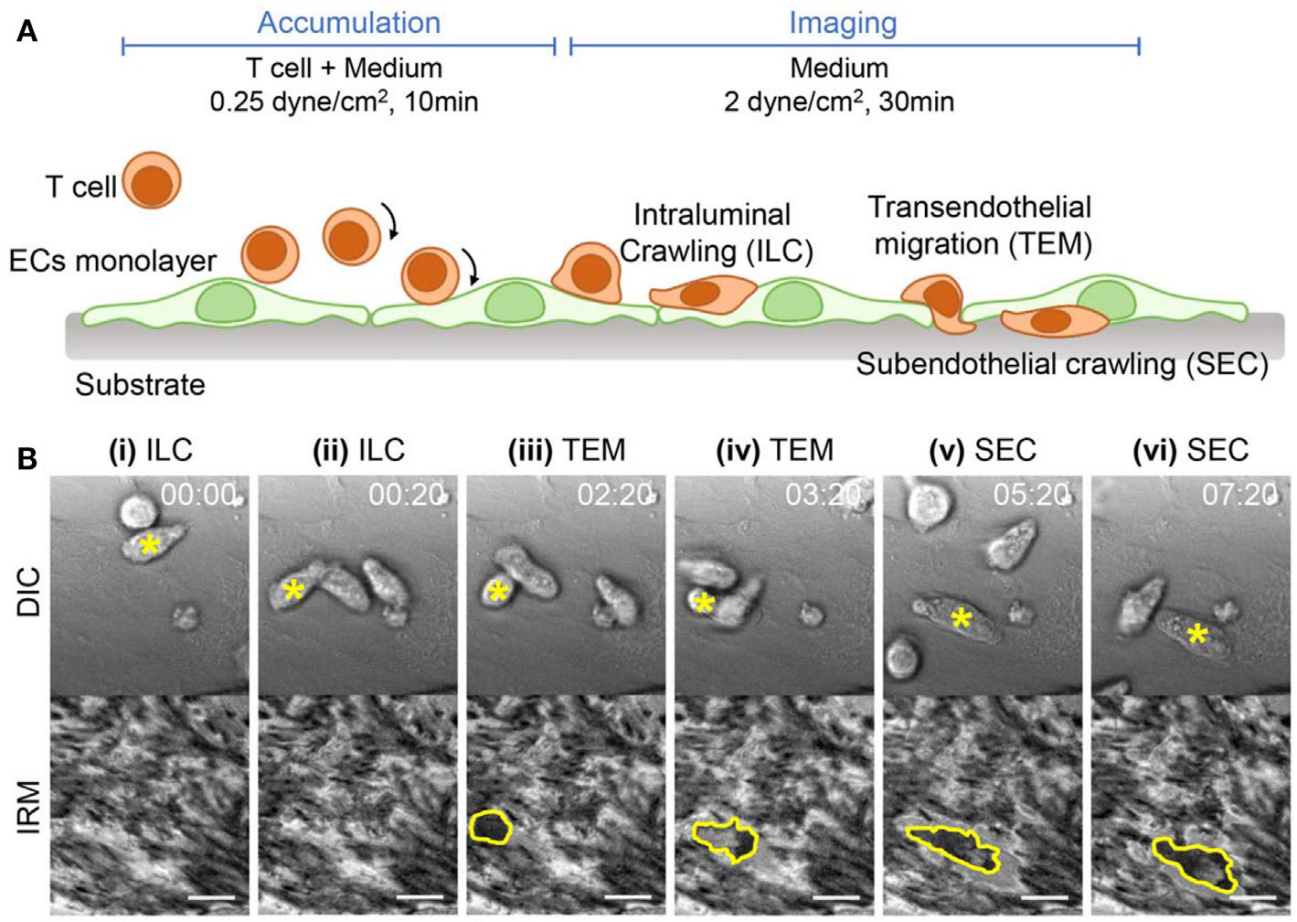
Experimental setup and time-lapse images of T cell adhesion cascades on EC monolayer. **(A)** Schematic illustration of experimental procedures and expected T cell dynamics. **(B)** Representative time-lapse differential interference contrast (DIC) (upper) and IRM images (lower). A T cell undergoing transitions of intraluminal crawling (ILC) → transendothelial migration (TEM) → subendothelial crawling (SEC) was marked with a yellow star in each DIC image, and the boundaries of the T cells appeared in IRM images were marked with yellow lines. Scale bar: 10 µm, elapsed time: mm:ss.

Next, we examined how EC adhesion influences T cell adhesion cascades for extravasation, in particular TEM and SEC steps. To visualize T cell–EC adhesion interactions during T cell extravasation, time-lapse imaging of differential interference contrast (DIC) and IRM images were simultaneously acquired with 20 s intervals for 30 min under flow experiment setting, schematically shown in Figure 3A. Using these two distinct types of images, we could clearly monitor T cell trans-migration processes across EC monolayer (Figure 3B; Video S1 in Supplementary Material). In DIC images, a T cell performing ILC on EC monolayer was clearly visible with dark shadows at the peripheries (top panels of Figures 3Bi,ii), whereas the T cell underneath EC monolayer performing SEC lacked the dark shadows at the peripheries (top panels of Figures 3Bv,vi). In IRM images, by contrast, the T cell on EC monolayer was not visible (bottom panels of Figures 3Bi,ii), but the T cell underneath EC monolayer was clearly visible by dark signals (bottom panels of Figures 3Bv,vi). T cells in subendothelial spaces are tightly confined between ECs and glass coverslips, thus likely to have close contact with coverslips. For the T cell undergoing TEM, one part of a T cell on EC monolayer exhibited dark shadow in the DIC images (top panel of Figures 3Biii,iv) whereas the other part of the T cell underneath EC monolayer was shown as dark spots in the IRM images (bottom panel of Figures 3Biii,iv). Importantly, the EC monolayer itself generated distinct patterns in IRM images reflecting their adhesion patterns (Figure 1A), thus IRM imaging alone might not be sufficient to distinguish T cells underneath EC monolayer from EC adhesions. Taken together, by combining information on DIC and IRM images, we could simultaneously monitor T cells performing ILC, TEM, and SEC and EC adhesions in label-free fashions.

### TEM Preferentially Occurred at Sites Where NJ-FA Density Was Low

**Figure 4:**
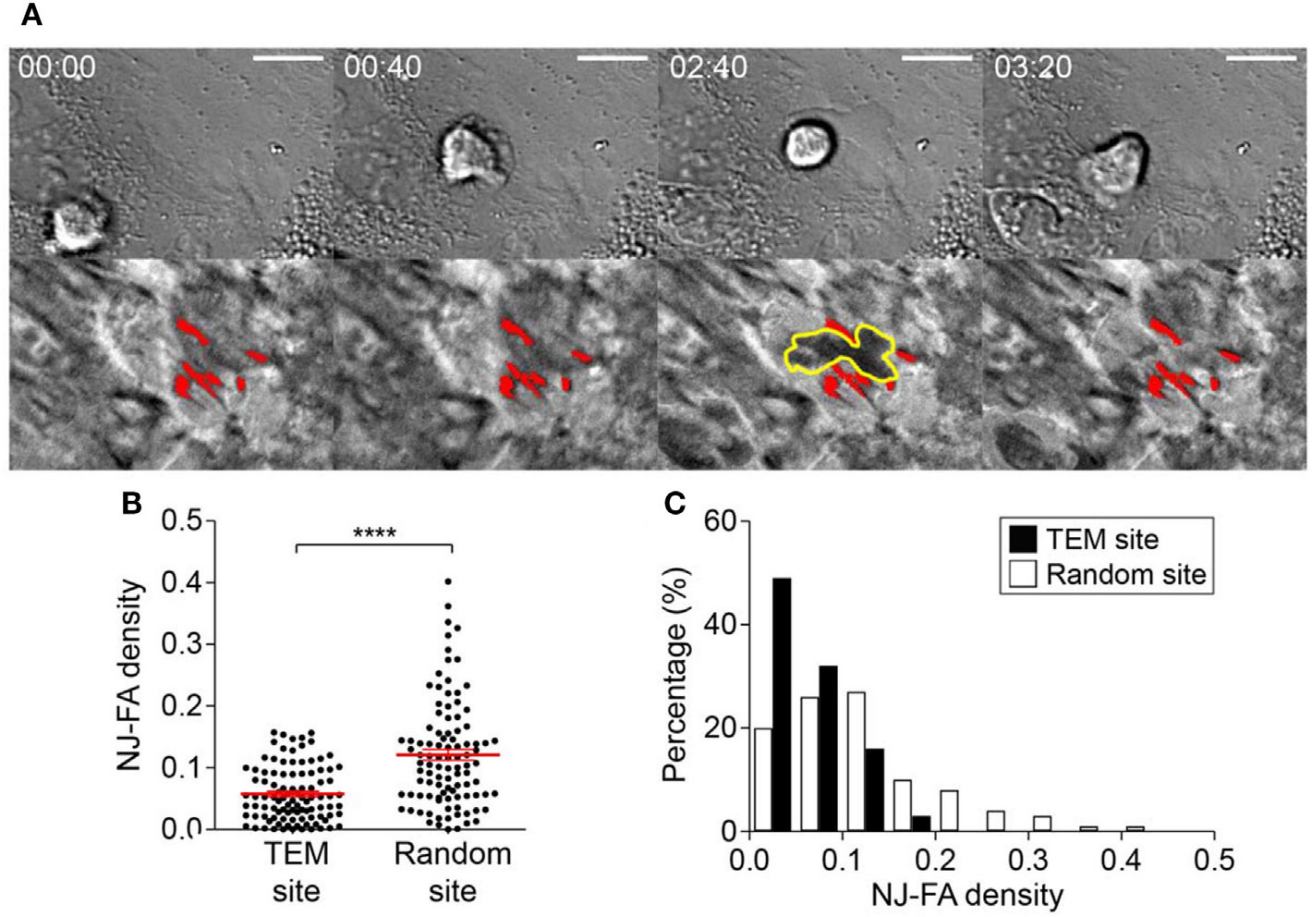
NJ-FA density of EC monolayer is a critical factor for successful transendothelial migration (TEM) of T cells. **(A)** Representative time-lapse images of T cells failed to proceed TEM after encountering high density EC-FA clusters. Yellow lines in IRM: boundary of a T cell underneath ECs; red spots in IRM: EC-FAs encountered by the T cell. Scale bar: 10 µm, elapsed time: mm:ss. **(B)** NJ-FA density of randomly selected sites or the sites where TEM occurred (Mann–Whitney *U*-test, ****p < 0.0001). **(C)** NJ-FA density distribution of TEM or randomly selected sites (two-sample Kolmogorov–Smirnov test, *p* < 0.0001).

As a first step for TEM, T cells extended protrusions into EC junctions to access subendothelial spaces (Figure 3Biii), and it was likely that EC-FAs interfered T cells access to subendothelial spaces. Indeed, we observed that some T cells undergoing TEM retracted pseudopods in subendothelial spaces back to apical surface of ECs when pseudopods encountered EC-FAs (11 out of 40; Figure 4A; Video S2 in Supplementary Material). Therefore, local density of EC-FAs in NJ regions might be an important factor for determining successful sites for TEM. To test this possibility, we measured NJ-FA density of the junctions where T cells successfully performed TEM, and compared it with NJ-FA density of randomly selected junctions (Figures 4B,C). Clearly, ~80% of TEM occurred at junctions with NJ-FA density <0.1, and no TEM was observed if NJ-FA density >0.2. By contrast, NJ-FA density of randomly selected sites exhibited median value >0.1, and 17% of sites exhibited NJ-FA >0.2. These results suggested that NJ-FA of ECs acted as physical barriers for T cells undergoing TEM, thus TEM preferentially occurred sites with low NJ-FA density.

### EC-FAs Guided T Cell SEC

**Figure 5:**
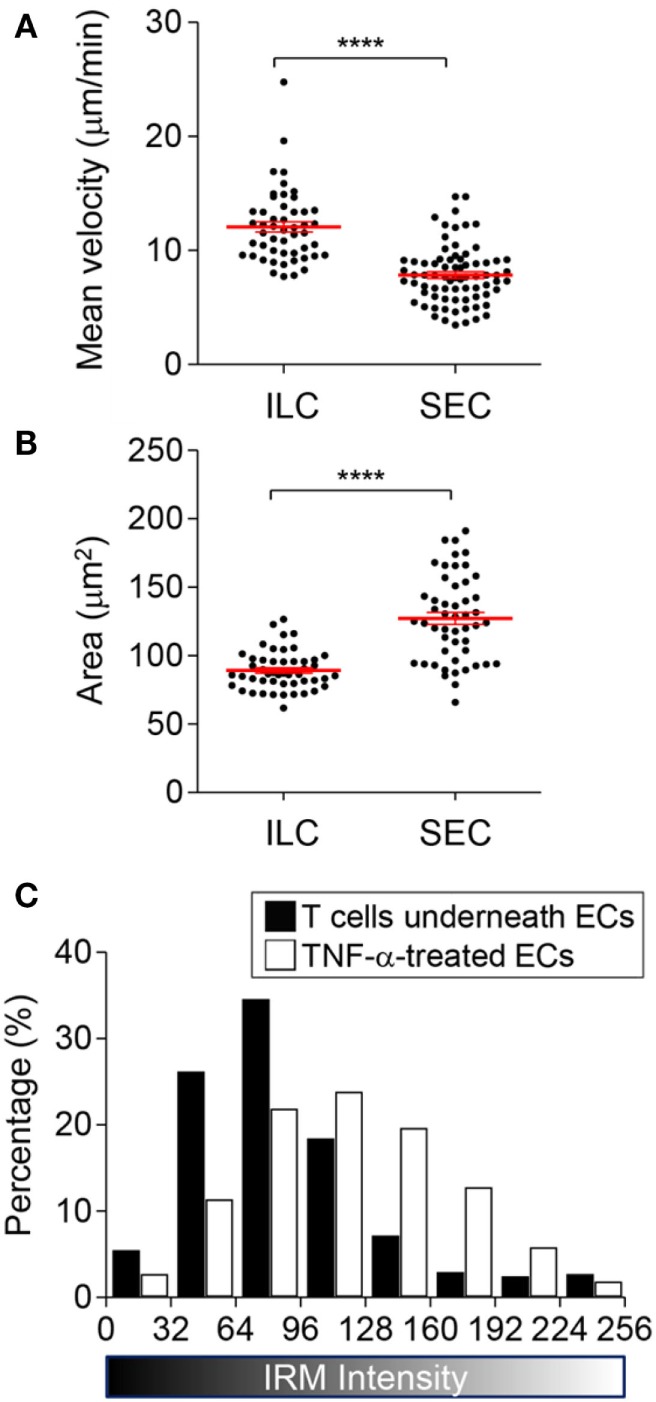
Characteristics of T cells undergoing subendothelial crawling (SEC). **(A,B)** Mean velocity **(A)** and area **(B)** of T cells undergoing intraluminal crawling (ILC) vs. SEC (Mann–Whitney *U*-test, ****p < 0.0001). **(C)** IRM intensity distribution of T cells undergoing SEC in comparison with TNF-α treated ECs (two-sample Kolmogorov–Smirnov test, *p* < 0.0001).

Next, we characterized T cells performing SEC. Compared with T cells conducting ILC, T cells performing SEC exhibited larger projected areas with slower migration velocity (Figures 5A,B). These results indicated that T cells underneath EC monolayer experienced substantial resistance for their crawling because they were tightly confined between basal part of ECs and glass substrate. Indeed, T cells under EC monolayer exhibited narrow IRM intensity distribution with low average intensities, meaning substantial fraction of basal membranes of T cells made tight contact with the substrate (Figure 5C).

**Figure 6:**
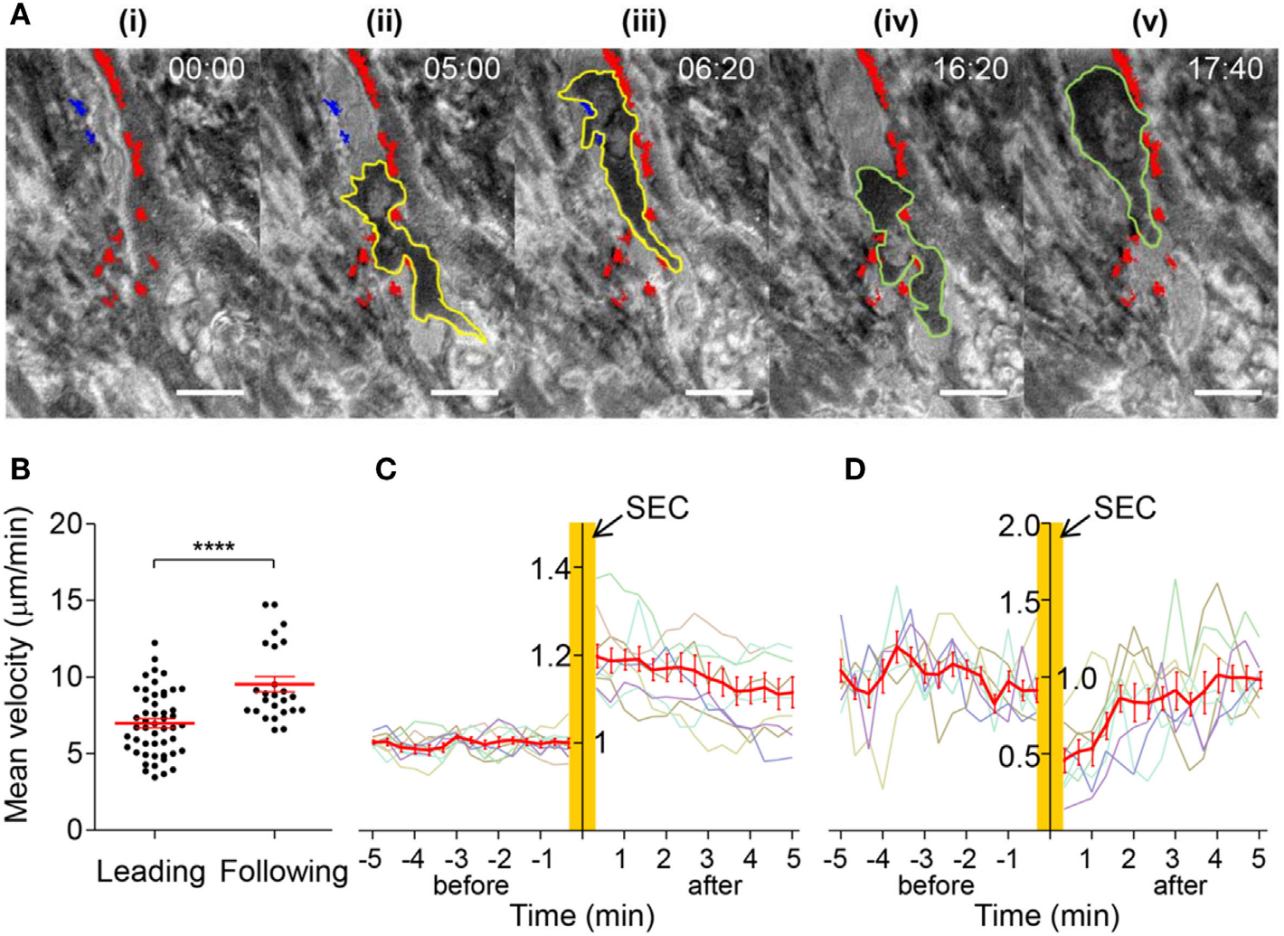
Dynamics of T cells undergoing SEC. **(A)** Representative time-lapse IRM images of T cells undergoing SEC. Yellow lines: boundary of a leading T cell; green lines: boundary of a following T cell; red spots: stable FAs encountered by T cells; blue spots: detached FAs by adjacent T cells undergoing SEC. Scale bar: 10 µm, elapsed time: mm:ss. **(B)** Mean velocity of leading or following T cells (Mann–Whitney *U*-test, ****p < 0.0001). **(C)** Normalized IRM intensity of EC monolayer before and after SEC of T cells. IRM intensity was normalized with average IRM intensity of EC monolayer before SEC of T cells. Normalized IRM intensity of nine individual regions vs. time (min) was plotted with gray lines, and the average of normalized IRM intensity in each time was plotted with a red line. **(D)** Normalized EC-FA size before and after SEC of T cells. EC-FA area was normalized with average EC-FA area before SEC of T cells. Normalized EC-FA area of eight individual EC-FAs vs. time (min) was plotted with gray lines, and the average of EC-FA area in each time was plotted with a red line.

Detailed IRM image analysis revealed that EC-FAs restricted morphologies and motilities of T cells in subendothelial spaces. In other words, T cells underneath the EC monolayer rarely broke FAs during their SEC (9 FAs were detached out of 50 FAs encountered by T cells; for example: blue spots in Figure 6A; Video S1 in Supplementary Material). As a result, T cells conducting SEC appeared to share common pathways underneath EC monolayer defined by undetached EC-FAs (Figure 6A, red spots; Video S3 in Supplementary Material), and different T cells under similar locations exhibited similar morphologies (yellow boarder line in Figure 6Aii and green boarder line in Figure 6Aiv). Interestingly, velocity of the “leading” T cells was significantly lower than “following” T cells (Figure 6B), indicating “leading” T cells somehow modified subendothelial spaces to facilitate migration of “following” T cells. We hypothesized that “leading” T cells substantially deformed cytoplasm of ECs so that subendothelial spaces formed between basal part of ECs and glass substrate would be widen by SEC of T cells. To test this hypothesis, we monitored IRM signals of areas where T cells passed by SEC. IRM intensities of EC monolayer before SEC exhibited minimal fluctuation. Immediately after SEC, IRM intensities of basal part of EC monolayer substantially increased, ~20% on average, and gradually decreased over time (Figure 6C). This result indicated that SEC of T cells widen subendothelial spaces by substantially deforming EC cytoplasm, and deformed EC cytoplasm slowly recovered to the original positions, exhibiting viscoelastic behaviors (38). SEC of T cells also significantly reduced neighboring FA areas down to 50% of original areas, but FA size recovered to the original one within 2 min after SEC (Figure 6D). Taken together, T cells substantially deformed EC cytoplasm and temporarily weakened EC-FAs during SEC. Importantly, only few EC-FAs were completely detached and the majority of EC-FAs only transiently weakened during SEC, presumably to widen subendothelial spaces, but quickly recovered the original adhesion areas. Therefore, similar to TEM, EC-FAs acted as physical barrier for T cells underneath ECs, or EC-FAs predetermined pathways of T cell SEC.

## Discussion

In this study, we examined detailed dynamic interactions between T cells and EC adhesions by employing IRM, which is a specialized imaging technique for label-free imaging of cell–substrate interactions. IRM has been mostly used to study adhesion dynamics of individual cells (23–25, 28), and as far as we know, this is the first study utilizing IRM to observe dynamics of one type of cells underneath the other type of cells. To achieve this goal, we first compared IRM images of EC monolayer in the absence of T cells with IFM images of EC-FAs (vinculins), and identified that dark spots in IRM images matched well with EC-FAs in IFM images (Figure 1). Then, by acquiring time-lapse IRM and DIC images simultaneously and comparing them, we could distinguish EC adhesions and T cells underneath EC monolayer in IRM images (Figure 3). In this way, we could observe detailed dynamic interactions between T cells and EC-FAs during TEM (Figure 4) and SEC (Figure 5) in a label-free manner. Similar observation of EC-FAs could have been made using fluorescence live cell imaging using ECs expressing FA-associated proteins, including paxillin, zyxin, and vinculin, fused with GFP. However, IRM imaging has several advantages compared with fluorescence imaging of FA-associated proteins. First, for fluorescence imaging of FA-associated proteins, ECs need be transfected to express GFP-fused proteins, which is laborious. Moreover, transfection efficiency of ECs is low, typically <50% (39), thus monolayer-level observation of EC-FAs is technically challenging. Second, live cell imaging of fluorophores can cause photobleaching and phototoxicity, which will limit observation duration or time resolution, and complicate data analysis (40, 41). Third, most importantly, IRM provides semi-quantitative information about cell membrane–substrate distance as well as FAs.

EC activation by pro-inflammatory cytokines such as TNF-α is a key initial step for leukocyte extravasation (3, 4, 35, 42). Phenotypes of the cytokine-activated ECs have been extensively characterized: they increase cytokine/chemokine production (35, 36) and surface expression of adhesion molecules (30–32), and contract entire of cell body through actomyosin cytoskeletons to loosen junctions between ECs (33, 34). So far, most studies primarily focused on changes in apical and lateral parts of ECs that influences earlier steps of the leukocyte adhesion cascade, including rolling, arrest, ILC, and TEM, and relatively little attention has been paid to basal part of ECs that comprise subendothelial spaces. In this study, we found that TNF-α-activated ECs also alters adhesion patterns of ECs, which substantially remodel subendothelial spaces to facilitate TEM and SEC of leukocytes. TNF-α-activated ECs exhibited significantly higher IRM intensity than untreated ECs (Figure 2A), meaning TNF-α-treatment increased distance between EC membrane and substrate to widen subendothelial spaces. Considering that T cells performing SEC exhibited tightly squeezed morphologies with slower migration velocity than T cells undergoing ILC (Figures 5A,B), and that widening subendothelial spaces by the leading T cells significantly enhanced migration velocity of the following T cells (Figure 6B), widening subendothelial spaces by TNF-α-treatment would also facilitate SEC of leukocytes. In addition to widening subendothelial spaces by weaken adhesions, we demonstrated that NJ-FA density of ECs was substantially decreased by TNF-α-treatment (Figure 2B). Altered FA distribution indeed facilitated TEM because NJ-FAs acted as physical barriers for TEM, and TEM preferentially occurred through the sites where NJ-FA density was low (Figure 4). Taken together, TNF-α-mediated EC activation substantially remodel subendothelial spaces to facilitate leukocyte TEM and SEC.

EC monolayer cultured on thin glass coverslips used in this study enabled us to visualize detailed dynamic interactions between EC adhesions and T cells underneath EC monolayer by IRM. However, subendothelial spaces in our study are much simpler than *in vivo* subendothelial spaces, thus our results need to be carefully interpreted. *In vivo*, an EC layer is located on a pericyte/basement membrane layer. Confocal analysis of basement membranes of post capillary venules performed by Nourshargh group showed the existence of LERs, where expression levels of ECM components are low (17). Importantly, LERs align well with tri-cellular junctions of EC layers where leukocyte TEM frequently occurs (16). Since LERs contain less amounts of ECM components such as type IV collagen and laminins, supporting integrin-mediated adhesion, than other regions of basement membranes, we speculate EC-FA density on LERs is also low. Therefore, frequent leukocyte TEM through EC layers on LERs of the basement membranes is likely to have the enhanced accessibility of subendothelial spaces by leukocytes due to low NJ-FA density. Intravital imaging revealed that neutrophils in subendothelial spaces preferentially crawled along pericytes and eventually breached LERs of basement membranes (18). Interestingly, SEC behaviors of neutrophils observed in intravital imaging and those of T cells observed in our study share some common features: they exhibited flattened morphologies indicating that leukocytes in subendothelial spaces were confined in between EC layers and underlying substrate, either thin glass coverslips or basement membranes/pericytes. In addition, multiple cells followed the same tracks, and the frontier cells were slower than following cells. In our experimental setting, subendothelial spaces are formed in between EC monolayer and glass coverslip. Our previous study (13) demonstrated SEC of T cells was mediated by LFA-1-dependent adhesion on ICAM-1 expressed on basal part of ECs, as anti-LFA-1-treatment ceased SEC of T cells. Leading cells substantially deformed EC cytoplasm, widening subendothelial spaces, and deformed EC cytoplasm exhibited viscoelastic behavior, thus slowly recovered to the original position. During viscoelastic recovery of EC cytoplasm, following cells could migrate through wider subendothelial spaces than the leading cells did, which could facilitate migration of the following cells. Migratory tracks were determined by EC-FAs and nuclei, which was substantially stiffer than cytoplasm so that T cells could not deform during SEC (13). By contrast, *in vivo* subendothelial spaces are formed in between EC layers and pericytes/basement membrane and SEC of neutrophils was mediated by LFA-1/Mac-1. Importantly, neutrophils exclusively crawled on pericytes, and chemokines and ICAM-1 expressed on pericytes were likely to be major factors guiding SEC of neutrophils. However, considering neutrophils crawling on pericytes shared common pathways and exhibited similar behaviors as our study, biophysical cues identified in our study such as FAs and viscoelasticity of cytoplasm may also play important roles in regulating SEC of leukocytes *in vivo*. In other words, adhesions formed between pericyte–basement membrane and pericyte–EC may restrict leukocyte migration on pericytes, and viscoelastic deformation of EC and pericyte cytoplasm caused by SEC of the leading leukocyte may transiently widen subendothelial spaces to facilitate SEC of the following leukocytes.

## Materials and Methods

### Cell Preparation

A EC monolayer was formed by culturing bEnd.3 cells (mouse brain endothelial cells, ATCC) on gelatin-coated coverslips. Coverslips (diameter: 18 mm, Marienfeld) treated with air plasma (200–500 W, Femto Science, Korea) for 1.5 min were placed in wells of a 12-well plate and incubated with 0.1% gelatin solution (Sigma) for 30 min at 37°C for coating. bEnd.3 cells (10^5^ cells/well) in DMEM medium containing 10% FBS (Gibco) and 1% penicillin–streptomycin (Invitrogen) were seeded on the gelatin-coated coverslips and cultured for 48 h in an incubator maintaining 37°C of temperature and 5% of CO_2_.

DO11.10 T blasts (T cells) were prepared from DO11.10 T cell receptor transgenic mice (Jackson Laboratories) bred in POSTECH Biotech Center (PBC). All experiments regarding mice were approved by the Institutional Animal Care and Use Committee at PBC. On day 0, cells in lymph nodes and spleens of DO11.10 mice were isolated and stimulated with 1 µg/ml of OVA323–339 peptides (ISQAVHAAHAEINEAGR, Peptron, Inc., Korea) in RPMI 1640 medium (Invitrogen) containing 10% of FBS, 1% penicillin–streptomycin, and 50 µM of beta-mercaptoethanol (Sigma). On day 2, 5 ng/ml (1–2 U/ml) of IL-2 was added. Cells on day 5 were used in all experiments.

### Fluorescence Microscopy and Interference Reflection Microscopy (IRM)

A modified Zeiss Axio Observer.Z1 epi-fluorescence microscope with a 40× (Plan-Neofluar, NA = 1.3) objective lens and a Roper Scientific CoolSnap HQ CCD camera were used for imaging. XBO 75 W/2 Xenon lamp (75 W, Osram) and DAPI (EX. 365, BS 395, EMBP 445/50), GFP (EX BP 470/40, BS 495, EMBP 525/50) filter sets were used for fluorescence imaging. For IRM, fluorescence filters were replaced with a linear polarizer, a narrow band-pass filter (EX BP 633/10), a beamsplitter (20/80) and a crossed analyzer. The microscope was automatically controlled using Axiovision 4.6 (Carl Zeiss). The acquired images were analyzed and processed using ImageJ (NIH).

### Shear Chamber Assay

A EC monolayer was stimulated with TNF-α (10 ng/ml) for 4 h, incubated with SDF-1α (100 ng/ml) for 10 min, and mounted on a shear chamber (Chamlide CF, Live Cell Instrument, Korea) with channel dimensions of 0.2 mm (height), 2 mm (width), and 17 mm (length). DO11.10 T cells (2 × 10^6^ cells/ml) suspended in growth media were perfused over the EC monolayer using a syringe pump (New Era Pump Systems, US) directly connected to the inlet of the shear chamber. A stage heater (Live Cell Instrument, Korea) was used to maintain a constant temperature of 37°C during experiments. T cells were first perfused at 0.25 dyne/cm^2^ of shear stress for 10 min to accumulate T cells on activated bEnd.3 EC monolayer. Then, shear stress was elevated to 2 dyne/cm^2^ for 30 min by perfusing culture media. The dynamics of T cells in the flow chamber was observed by time-lapse imaging with 20 s interval for 30 min.

### Immunostaining

To visualize FAs of ECs, ECs were fixed with 4% paraformaldehyde for 15 min at room temperature (r.t.), washed with PBS five times, permeabilized with 0.2% triton X-100 in PBS for 15 min at r.t., and washed with PBS five times. Then, fixed ECs were immersed in blocking buffer (2% FBS, 0.1% sodium azide, and 1 mM EDTA in PBS) for 30 min at r.t. and stained with sequential treatment of anti-vinculin antibody (polyclonal, Invitrogen, 0.2 µg/ml in blocking buffer) overnight at 4°C and Alexa Fluor 488-conjugated secondary antibody [F(ab′)2-Goat anti-Rabbit IgG (H + L), polyclonal, eBioscience, 2 µg/ml in blocking buffer] for an hour at r.t. To visualize adherens junction, anti-VE-cadherin-Alexa Fluor 488 (clone: eBioBV13, eBioscience, 0.5 µg/ml in blocking buffer) was added to ECs after paraformaldehyde fixation and incubated for an hour at r.t.

### Statistical Analysis

The statistical significance was tested using the Mann–Whitney *U*-test or Kolmogorov–Smirnov test. For bar graphs, average values with standard error of mean (SEM) are presented.

## Ethics Statement

All experiments regarding mice were approved by the Institutional Animal Care and Use Committee at POSTECH Biotech Center (PBC).

## Author Contributions

JL, KHS, and JD designed the study and developed the methodology. JL performed and analyzed all the experiments. TK assisted image processing. JL and JD wrote the manuscript.

## Conflict of Interest Statement

The authors declare that the research was conducted in the absence of any commercial or financial relationships that could be construed as a potential conflict of interest.

## Supplementary Material

Video S1 A representative T cell on endothelial cell monolayer proceeding intraluminal crawling (ILC)–transendothelial migration (TEM)–subendothelial crawling (SEC) transitions. Scale bar: 10 µm, elapsed time: mm:ss. Left panel: differential interference contrast (DIC); right panel: interference reflection microscopy (IRM).

Video S2 A representative T cell retracting its pseudopod in subendothelial space back to luminal surfaces after encountering endothelial cell focal adhesions. Left panel: differential interference contrast (DIC); right panel: interference reflection microscopy (IRM).

Video S3 Representative T cells undergoing subendothelial crawling. Left panel: differential interference contrast (DIC); right panel: interference reflection microscopy (IRM).
